# The Delicate Dance of Intraoperative Anesthesia: Addressing Patient and Anesthesiologist Concerns

**DOI:** 10.7759/cureus.54746

**Published:** 2024-02-23

**Authors:** Zaid Al Modanat, Lou'i Al-Husinat, Bashar M Mistarihi, Mohammad Tashtoush, Jood Alsarabi, Rama Matalqah, Hassan Mistarihi, Mohammad Wasfi Amir, Nawal Debajah, Esra’a Rejoub, Raneem Bereshy, Mustafa Tawaha, Rana Talj, Giustino Varrassi

**Affiliations:** 1 Department of Clinical Sciences, Faculty of Medicine, Yarmouk University, Irbid, JOR; 2 Department of Clinical Medical Sciences, Faculty of Medicine, Yarmouk University, Irbid, JOR; 3 Department of Neurology, Faculty of Medicine, Yarmouk University, Irbid, JOR; 4 Department of Anesthesiology, Faculty of Medicine, Yarmouk University, Irbid, JOR; 5 Department of Obstetrics and Gynaecology, Faculty of Medicine, Yarmouk University, Irbid, JOR; 6 Department of General Surgery, Faculty of Medicine, Mutah University, Karak, JOR; 7 Internal Medicine, Rochester General Hospital, New York, USA; 8 Pain Medicine, Paolo Procacci Foundation, Rome, ITA

**Keywords:** doctor-patient relationship, patient-physician relationship, concerns, psychology, anesthesia

## Abstract

Background

In the realm of surgical procedures, patients and anesthesiologists have distinct concerns that can have an impact on their relationship. Patients are often riddled with anxiety about the unknowns of anesthesia and the possible risks. Anesthesiologists, too, face their own set of concerns. Despite the importance of this interaction, there has been little research on the specific concerns of both parties. Our study aims to fill this gap by describing and comparing the concerns of patients and anesthesiologists in Jordan.

Methodology

This cross-sectional study evaluated anesthesia-related problems based on specific questionnaires. The responses to the questionnaires were on a voluntary basis. The consent of the participants was granted after the aims of the study were clarified. Data were collected and analyzed using SPSS version 28 (IBM Corp., Armonk, NY, USA).

Results

A total of 155 Jordanian anesthesiologists and 1,858 participants from the population who had undergone anesthesia participated in the study. In general anesthesia, over 60% of the anesthesiologists were most worried about ventilation and intubation difficulties during anesthesia induction and death at the end of anesthesia. Regarding regional anesthesia, the primary concerns included toxicity from local anesthesia infiltration (64.5%) and total spinal anesthesia (49.0%). Patients were concerned about various anesthesia-related scenarios, with the highest worries about pain (3.41/4), a sharp drop in vital signs (3.40/4), and an irregular heartbeat (3.39/4). Female patients, those with lower incomes, and those with a bachelor’s degree reported higher anesthesia concern levels. Additionally, anesthesiologists’ mean concern score was significantly lower than that of patients.

Conclusions

Patients concentrated on pain, a drop in vital signs, and irregular heartbeats, whereas anesthesiologists were worried about ventilation, intubation, and hypoxia. Patients placed more emphasis on personal experiences and social factors than technical issues. Therefore, patient education about anesthesia and discussion about intra and postoperative expectations are imperative to improve the surgical experience and the relationship between patients and anesthesiologists.

## Introduction

In the world of surgical procedures, both patients and anesthesiologists have unique concerns that might negatively affect the relationship between them pre and intraoperatively. This relationship is very important to ensure successful outcomes. Anxiety tends to run high during this time, with patients often expressing concerns about the risks associated with their operation [1]. This sentiment is supported by the findings of an extensive observational study, which revealed that anxiety was the most frequently cited concern among over 15,000 non-obstetric surgery patients [2]. In a cross-sectional study by Aust et al. (2018), 92.6% of adults expressed preoperative anxiety using the Amsterdam Preoperative Anxiety and Information Scale [3]. Moreover, the COVID-19 pandemic has introduced a new layer of anxiety for patients, as they now consider the risk of airborne pathogens when they undergo a surgical procedure [4].

Eberhart et al. [1] described a great example of how specific fears that patients undergoing elective surgery harbor may affect the operation. Moreover, the literature is abundant with possible complications that raise concerns for anesthesiologists [5-7].

There is limited literature addressing the level of concern between patients and anesthesiologists, nonetheless, comparing the similarities and differences between them. Therefore, our study aims to describe and compare patients’ and anesthesiologists’ concerns among Jordanians, hoping for a better understanding and communication between the two parties.

This article was previously posted to the Research Square preprint server on October 6, 2023.

## Materials and methods

A descriptive, cross-sectional, correlational study design was adopted. A self-administered questionnaire was distributed to 155 Jordanian anesthesiologists working in public, private, and university hospitals in Jordan, along with 1,858 volunteers from the general population who had undergone anesthesia within the past five years regardless of their age and were able to read, use social media, and answer the questionnaire. All participants received a personal cellphone message with the survey link. We asked participants from the general public and anesthesiologists to share the link to our questionnaire with others using a snowballing approach. The study was conducted voluntarily, and consent was obtained before starting the questionnaire. The confidentiality of the study participants’ information was guaranteed. Institutional Review Board (IRB) approvals were obtained from Yarmouk University (approval number: IRB/2023/138). This study was performed in accordance with the Declaration of Helsinki. Individuals with established psychiatric illnesses who were unable to converse and individuals who were illiterate in Arabic or English were excluded. Moreover, the children whose parents declined to participate or were unable to access an electronic copy of the survey due to technological limitations were also excluded from the study. Our study included two self-administered questionnaires, one for anesthesiologists and the other for the general population. An extensive literature review was conducted to retrieve relevant questions related to anesthesiology concerns, some of the questions were added based on our experiences and to be culturally competent. A first draft of both questionnaires was sent to a panel of experts that included five experts in anesthesiology; those with academic positions; and consultant anesthesiologists for face validity to provide their opinion regarding the clarity, simplicity, and relevance of the items. The survey was written in English and then professionally translated into Arabic, which is the native language of the general public in Jordan. However, it was distributed in the English language among anesthesiologists. A pilot study was conducted to test the study tool’s internal consistency, and Cronbach’s alpha coefficients were found to be 0.75 and 0.77, respectively. Both study tools were composed of the following two sections: the first section asked about the participants’ sociodemographic characteristics, and the second section asked about concerns regarding anesthesia. Concerns were rated on a four-point Likert scale, with higher scores reflecting higher concern.

Statistical analysis

Frequency and percentage were used to represent categorical data, while the mean and standard deviation were used to express the scale variables. Moreover, the scale scoring system was categorized into three levels (a score of 1-1.99 denoted a low level, 2-2.99 a moderate level, and 3-4 a high level). One-way analysis of variance (ANOVA) and independent t-tests were used to investigate the mean differences between the groups. A p-value <0.05 was deemed statistically significant. SPSS version 28 (IBM Corp., Armonk, NY, USA) was used to analyze the data.

## Results

Study findings for anesthesiologists

Participants’ Sociodemographic Characteristics

A total of 155 anesthesiologists were enrolled from February to March 2023. The majority were male physicians (127; 81.9%). Approximately half of the participants were in the age group of 25-34 years old (76; 49.0%). Most anesthesia physicians were working as anesthesia residents (66; 42.6%) (Table 1).

**Table 1:** Descriptive statistics for participants’ sociodemographic characteristics. *: Anesthesiologists who had finished their residency program but were not board-certified yet.

Variables	Category	Frequency	Percentage
Gender	Male	127	81.9
	Female	28	18.1
Physician’s age in years	25–34	76	49.0
	35–44	49	31.6
	45–54	16	10.3
	>55	14	9.0
Current position in anesthesia	Anesthesia resident doctor	66	42.6
	Anesthesia board-eligible doctor*	14	9.0
	Anesthesia attending/specialist	40	25.8
	Anesthesia consultant doctor (subspecialist or a specialist with >10 years of experience)	35	22.6

Level of Concerns Among Anesthesiologists Regarding Perioperative Care and Their Concerns While They Are Under Anesthesia

The anesthesiologists were instructed to rate 21 items on a four-point scale to reflect their level of perioperative concern and 15 items to demonstrate their anesthesia concern as if they were patients. The mean score for both concerns was calculated, and the results showed that their concern level was a mean score of 2.96 ± 0.71 for perioperative and a mean score of 2.92 ± 0.67 for anesthesiologists under anesthesia, demonstrating that they had a moderate-high concern on our four-point Likert scale.

Mean Difference of Anesthesiologists Regarding Perioperative Concern

To investigate whether there were statistically significant differences in perioperative concerns based on anesthesiologist characteristics, one-way ANOVA and independent t-tests were utilized, and the results in Table 2 demonstrate that perioperative concerns were not significantly different according to physician characteristics (p > 0.05 for all). In the same context, there were no statistically significant mean differences in anesthesiologist’s concerns during their time under anesthesia based on anesthesiologist sociodemographic characteristics (Table 2).

**Table 2:** Mean difference in anesthesiologists’ concerns regarding perioperative issues and their concerns while under anesthesia. t: independent t-test, F: one-way analysis of variance; p ≤ 0.05 indicates a statistically significant difference.

Variables	n	Mean ± SD	Test value	P-value	
Gender					Perioperative concerns (mean score 2.96 ± 0.71)
Male	127	2.99 ± 0.70	t = 1.401	0.163	
Female	28	2.79 ± 0.71			
Physician’s age in years					
25–34	76	3.05 ± 0.63	F = 1.199	0.312	
35–44	49	2.88 ± 0.78			
45–54	16	2.99 ± 0.79			
>55	14	2.71 ± 0.73			
Current position in anesthesia					
Anesthesia resident doctor	66	3.00 ± 0.74	F = 0.625	0.600	
Anesthesia board-eligible doctor	14	3.13 ± 0.72			
Anesthesia attending/specialist	40	2.86 ± 0.64			
Anesthesia consultant doctor (>10 years)	35	2.91 ± 0.73			
Gender					Concerns while under anesthesia (mean score 2.92 ± 0.67)
Male	127	2.94 ± 0.67	t = 1.067	0.288	
Female	28	2.79 ± 0.70			
Physician’s age in years					
25–34	76	3.00 ± 0.65	F = 1.029	0.381	
35–44	49	2.81 ± 0.69			
45–54	16	2.97 ± 0.75			
>55	14	2.77 ± 0.63			
Current position in anesthesia					
Anesthesia resident doctor	66	2.94 ± 0.68	F = 0.893	0.446	
Anesthesia board eligible doctor	14	3.07 ± 0.81			
Anesthesia attending/specialist	40	2.78 ± 0.63			
Anesthesia consultant doctor (>10 years)	35	2.96 ± 0.65			

*Anesthesiologists*’* Feared Complications During the Perioperative Period*

Anesthesiologists were most concerned about difficulties in ventilation and intubation during anesthesia induction (98; 63.2%), hypoxia during maintenance (61; 39.4%), and death at the end of anesthesia (63; 40.6%), as shown in Table 3.

**Table 3:** Percentages of complications that concern anesthesiologists perioperatively and concerns related to specific complications associated with local and regional anesthesia.

Concern type	Percentage	
During induction of anesthesia		Perioperative concern
Difficulty ventilation and intubation	63.2	
Vomiting and aspiration	20.6	
Hypotension and bradycardia	9.7	
Arrhythmia	4.5	
Allergic reaction	1.9	
During maintenance of anesthesia		
Hypoxia	39.4	
Arrhythmia	27.7	
Awareness	19.4	
Hypotension	9.0	
Hypertension	3.2	
Hypercapnia	0.6	
Hypocapnia	0.6	
At the end of anesthesia		
Death	40.6	
Respiratory complications	37.4	
Brain injury	12.3	
Cardiac injury	3.9	
Nerve injury	3.2	
Awareness	2.6	
Complication to the patient during local anesthesia infiltration		Concerns related to specific complications associated with local and regional anesthesia
Local anesthesia toxicity	64.5	
Failure of local anesthetic to provide sufficient anesthesia	15.5	
Infection	8.4	
Pain at the injection site	5.2	
Hematoma formation and bleeding	4.5	
Bruising at the injection site	1.9	
Complication to the patient during regional anesthesia		
IV injection of local anesthesia	31.6	
Permanent neurological injury	29.0	
Local anesthetic toxicity	19.4	
Block failure	14.8	
Vascular injury and bleeding	3.2	
Hematoma formation	1.9	
Complication to the patient during neuraxial anesthesia		
Total spinal anesthesia	49.0	
Respiratory failure from high spinal/block	18.1	
Hypotension	13.5	
Patchy block and failure of spinal/epidural	9.0	
Bradycardia	7.7	
Pain during needle insertion	1.3	
Nausea and vomiting	1.3	
Complication that might happen to patient after neuraxial anesthesia		
Post-dural puncture headache	27.7	
Nerve or spinal cord damage, possibly resulting in paralysis	19.4	
Spinal/epidural hematoma	18.7	
Spinal infection, including meningitis	14.2	
Direct spinal cord injury	7.7	
Epidural abscess	7.1	
Back pain	3.2	
Urinary retention	1.9	

*Anesthesiologists*’* Concerns Regarding Complications Related to Local and Regional Anesthesia*

Anesthesiologists’ primary apprehension pertained to the potential toxicity associated with local anesthesia infiltration (100; 64.5%). Moreover, 49 (31.6%) expressed concern about the inadvertent intravenous injection of local anesthesia. Among the complications related to neuraxial anesthesia, total spinal anesthesia accounted for the highest level of concern, accounting for 49.0% (n = 76). Lastly, postdural puncture headache was the most notable consequence after neuraxial anesthesia, evoking worry in 43 (27.7%) of the professionals surveyed (Table 3).

Anesthesiologists’ Concerns Toward Patient Characteristics and Comorbidities

**Figure 1:**
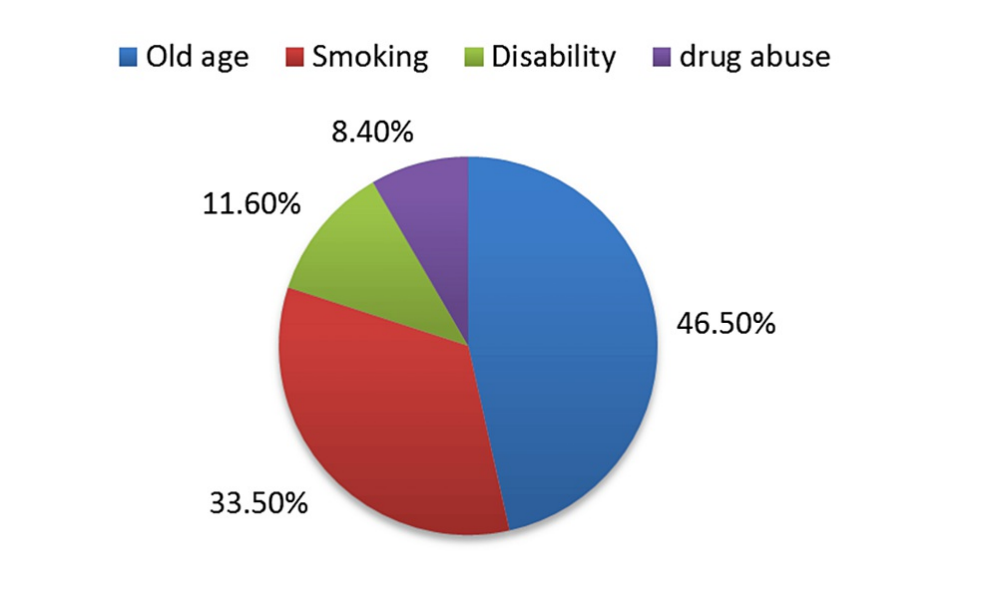
Anesthesiologists’ concerns about certain patient features based on patients’ characteristics.

**Figure 2:**
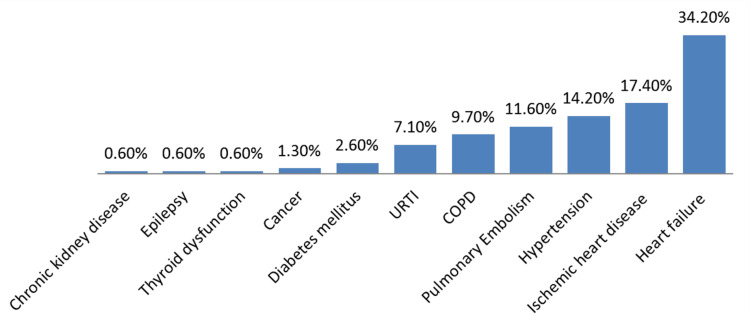
Anesthesiologists’ concerns toward certain patient features based on patients’ comorbidities. URTI = upper respiratory tract infection; COPD = chronic obstructive pulmonary disease

Old age, i.e., those above 60 years old, was the most concerning patient characteristic in 72 (46.5%) anesthesiologists (Figure 1). Figure 2 shows that heart failure was the most worrisome health condition in 53 (34.2%) of them. The least concerning comorbidities were chronic kidney disease, epilepsy, and thyroid dysfunction which were worrying only 1 (0.6%) of them, respectively.

Study results for patients

Participants’ Sociodemographic Characteristics

A total of 1,858 individuals were included in the study, of whom 1,183 (63.7%) were females, while 674 (36.3%) were males. One-third were between 18 and 24 years old; 990 (53.3%) were married; 1,319 (71.0%) were resident in cities; and 1,157 (62.3%) had a bachelor’s degree. Furthermore, the study revealed that 860 (46.3%) of participants earned between 1,000 and 2,000 JOD monthly, which is considered middle class according to the median income in Jordan. Participants were either students, employees, or retired; two-thirds of the employees worked outside the medical industry (Table 4).

**Table 4:** Participants’ sociodemographic characteristics (N = 1,858).

Variable	Category	Frequency	Percentage
Gender	Male	675	36.3
	Female	1,183	63.7
Age/years	<18	57	3.1
	18–24	574	30.9
	25–34	318	17.1
	35–44	393	21.2
	45–54	366	19.7
	55–56	117	6.3
	>64	33	1.8
Social status	Married	990	53.3
	Single	805	43.3
	Other	63	3.4
Place of residency	Urban	1,319	71.0
	Rural	539	29.0
Education level	Less than a high school	222	11.9
	Diploma	171	9.2
	Bachelor's degree	1,157	62.3
	Higher Degree	308	16.6
Monthly income/JOD	<1,000 (low income)	817	44.0
	1,000–2,000 (middle income)	860	46.3
	>2,000 (high income)	181	9.7
Occupation	Healthcare worker	378	20.3
	Nonhealthcare worker	769	41.4
	Student	431	23.2
	Retired	280	15.1

Patients’ Anesthesia Concern Level

On a four-point Likert scale, the patients were asked to rate how concerned they were on 23 different anesthesia-related items. According to Table 5, patients were most concerned about pain (mean = 3.41). Then there was the sharp alteration in vital signs (mean = 3.40), and an irregular heartbeat (mean = 3.39). The other items were less worrying. The findings additionally demonstrated that the patients’ total mean worry was 3.26 ± 0.67.

**Table 5:** Patient concern scores regarding different anesthesia situations.

Patients’ anesthesia concern	Mean ± SD	Patients’ anesthesia concern	Mean ± SD
Feeling of pain	3.41 ± 0.42	Receive improper care after anesthesia	3.28 ± 0.64
Sudden severe drop of vital signs and unable to resuscitate	3.40 ± 0.27	Becoming paralyzed due to anesthesia	3.27 ± 0.55
Irregular rate and cardiac arrest	3.39 ± 0.37	The need to be intubated during surgery	3.25 ± 0.64
Overdose of anesthesia	3.38 ± 0.57	Sore throat and dyspnea during surgery	3.21 ± 0.71
Awakening during surgery and feeling pain	3.37 ± 0.59	Headache	3.19 ± 0.78
feeling of being sick and discomfort	3.36 ± 0.61	Prolonged period in the intensive care unit	3.14 ± 0.67
Failure of general or epidural anesthesia	3.34 ± 0.49	Anesthesiologist leaving the operating room during the surgery	3.14 ± 0.56
Drop of oxygen saturation (hypoxia) and its complications	3.33 ± 0.55	Permanent damage to the brain tissue	3.14 ± 0.54
Heart attack and stroke during anesthesia	3.33 ± 0.61	Awakening while the tube is still in place	3.11 ± 0.44
Unable to wake up after anesthesia	3.31 ± 0.61	Getting harassed while unconscious	3.11 ± 0.52
Feeling nausea and vomiting	3.29 ± 0.45	Loss of memory	2.96 ± 0.55
Leaking of private information	3.29 ± 0.55	Overall mean concern score	3.26 ± 0.67

Patients’ Anesthesia Concerns Based on Their Sociodemographic Characteristics

The results shown in Table 6 revealed that female patients had significantly higher anesthesia concern mean scores than male patients (mean = 3.34 ± 0.54 vs. mean = 3.13 ± 0.61, p < 0.001). Additionally, the anesthesia concern score was significantly different based on the patients’ monthly income and educational level (p < 0.001 and p = 0.020, respectively). To demonstrate in favor of who the significant findings are related, the Tukey honest significant difference post hoc test for pairwise comparisons indicates that those with high income reported a lower anesthesia concern mean score than those with low and middle income (p < 0.001). No statistically significant difference in anesthesia concern was found between low- and middle-income individuals (p = 0.994).

**Table 6:** Mean differences in patients’ concerns based on their characteristics. Different superscript letters indicate significant differences (p < 0.05) in the same row. 1 USD is 0.71 JOD.

Variables	Categories	n	Mean ± SD	Test value	P-value
Patients’ gender	Male, Female	675, 1,183	3.13 ± 0.61, 3.34 ± 0.54	t = 7.686	<0.001
Patients’ monthly income/JOD	<1,000	817	3.28^a^ ± 0.47	F = 12.851	<0.001
	1,000–2,000	860	3.28^a^ ± 0.45		
	>2,000	181	3.09^c^ ± 0.63		
Patients’ educational level	Less than a high school certificate	222	3.17^a^ ± 0.48	F = 5.095	0.020
	Diploma	171	3.26^ab^ ± 0.66		
	Bachelor’s degree	1,157	3.31^b^ ± 0.66		
	Higher degree	308	3.18^ac^ ± 0.74		
Patients’ employment status	Inside of the medical field	378	3.23 ± 0.67	1.488	0.215
	Outside of the medical field	769	3.24 ± 0.71		
	Student	431	3.30 ± 0.63		
	Unemployed	280	3.31 ± 0.64		
Patients’ place of residency	Urban	1,319	3.25 ± 0.57	1.739	0.157
	Rural	539	3.29 ± 0.51		
Patients’ marital status	Married	990	3.25 ± 0.67	0.684	0.504
	Single	805	3.28 ± 0.66		
	Other	63	3.20 ± 0.69		
Patients’ age groups	<18	57	3.29 ± 0.66	1.281	0.262
	18–24	574	3.32 ± 0.63		
	25–34	318	3.24 ± 0.68		
	35–44	393	3.24 ± 0.67		
	45–54	366	3.22 ± 0.71		
	55–64	117	3.20 ± 0.73		
	>64	33	3.29 ± 0.50		

In the same context, bachelor’s degree holders demonstrated a higher anesthesia concern than those who had secondary and higher degrees (p = 0.018 and p = 0.010, respectively). No significant mean difference was noted between other educational levels. Furthermore, there were no significant mean differences (p > 0.05) for patients’ employment, place of residence, marital status, or age.

*Comparing the Mean Score of Anesthesiologists Under Anesthesia With That of Patients*’* Anesthesia Concerns*

A one-sample t-test was used to compare the mean concern score of anesthesiologists with that of patients, and the results indicated that the mean score of anesthesiologists was significantly lower than that of patients (2.92 vs. 3.26, t = 6.364, p = 0.001). Nonetheless, it is noteworthy that the difference in sample size between the two groups should be considered as the patient sample was approximately 12 times larger.

## Discussion

Anesthesia is a major specialty that allows important advancement in surgery, and there is a different public perception compared to other medical specialties [7]. Given that, concerns about anesthesia can vary from those of an anesthesiologist. This study aimed to gain insights into patients’ and anesthesiologists’ concerns about undergoing anesthesia. We also assessed complications due to anesthesia in the two groups. Considering how easily information can spread through the media, ideas and concerns about anesthesia can vary widely among the population.

The study’s findings that revealed patients’ top concerns were pain, a sudden drop in vital signs, and an irregular heartbeat, with mean scores of 3.41, 3.40, and 3.39, respectively. Comparing this to a systematic review of anesthesia education, common concerns included pain, death, intraoperative awareness, and postoperative nausea and vomiting [8]. The slight variations may be linked to educational levels, as seen in a Jordanian study on patient perceptions of anesthesia practice, where over half of the patients had limited knowledge about the anesthesiologist’s role [9].

On the other hand, anesthesiologists’ main concern during anesthesia induction was difficulties in ventilation and intubation. Fear of challenging intubation has been present since the birth of anesthesia. Nevertheless, it is still of most importance today. Therefore, multiple studies, in agreement with the concerns of anesthesiologists, have shown how difficult intubation and other risk factors, including obesity or dental problems, can increase the risk of anesthesia as well as surgical complications [10,11]. Furthermore, anesthesiologists use various methods to assess the patient’s airway and, based on the results, can adequately estimate the difficulty of intubation and whether an intervention is needed [12]. This further enforces how proper intubation is one of the cornerstones of anesthesia, making it among the biggest worries for anesthesiologists. Induction is simply the first step in anesthesia, and further potential complications can arise for the anesthesiologist during the maintenance phase and at the end of anesthesia. Our survey showed that the most feared complication during the maintenance phase was the development of hypoxia, while death was the most significant concern toward the end of anesthesia. Therefore, detailed and continuous perioperative monitoring is crucial to mitigate the occurrence of intraoperative complications.

The differences in concerns between the population and anesthesiologists highlight each group’s distinct perspectives regarding anesthesia. Anesthesiologists primarily focus on the technical aspects and potential complications associated with anesthesia. In contrast, the general population expresses concern about personal experiences, possible discomfort, and the influence of social media and community culture.

Pulmonary aspiration due to vomiting is the second most concerning complication to anesthesiologists during induction (20.6%; n = 32). Depending on risk factors, pulmonary aspiration complicates between 1 and 900 and 1 and 10,000 general anesthesia cases [13]. Eight out of 16 (50%) anesthesia-related deaths and 23 out of 133 (17%) reported that primary anesthesia events were caused by inhalation [14]. This has always been a major concern due to the potential for serious complications such as inhalation pneumonia and respiratory complications [15,16]. This offers a potential explanation for the increased level of concern surrounding this complication in our study.

Limitations

The inclusion of older literature is one of the limitations; studies used in our research were mostly conducted more than five years ago, and as a result, new developments or changes in the field may not have been appropriately taken into account. Despite this limitation, it should be acknowledged that the selection of studies in the literature was based on the relevance and availability of data. However, the inclusion of older data provided a historical backdrop and a thorough survey of the body of literature. An in-depth literature review to look for any significant developments in this field was done to address this limitation. Additionally, using social media for distributing the questionnaire introduced a second limitation as some participants might have misinterpreted instructions or questions, or were unable to complete the survey in full leading to fewer responses. Third, only 3% (n = 56) of our responses were patients aged 18 and less, while we acknowledge that this age category is very broad, and may include a sample who may not grasp questions regarding anesthesia, however; we anticipate that those who responded are likely not in a very young age group, considering their ability to understand and answer the questions in the questionnaire, and by including this age group we have a better representation of the general population. Fourth, recall bias is one of the limitations of our study. To mitigate this, responders who underwent anesthesia more than five years ago were excluded to minimize recall bias.

Strengths and implications

Up until now, no paper has combined the perspectives of both the patient and the anesthesiologist regarding anesthesia. Knowing the concerns of patients in anesthesia and comparing them to those of anesthesiologists will help in establishing effective patient-physician communication in which the patient feels less anxious and makes it easier for the physician to do their job in an environment in which they are trusted and result in a lower incidence of malpractice litigation. We look forward to leveraging our research findings for the development and implementation of educational programs and communication strategies aimed at addressing the specific concerns identified in the study and assessing their effectiveness.

## Conclusions

Addressing patients’ and anesthesiologists’ concerns regarding anesthesia is our study’s main aim. Our research is the first to address the differences in anesthesia concerns between both groups. Patients were concerned about pain, drops in vital signs, and irregular heartbeat. Anesthesiologists were concerned about difficulties in ventilation and intubation, hypoxia, and death. These concerns can be minimized by enforcing patient education in the preoperative period in addition to improving intubation techniques, along with using video-assisted intubation. For this purpose, awareness campaigns for the general public and continual education and training for anesthesiologists can be helpful.
